# Vitamin A cycle byproducts impede dark adaptation

**DOI:** 10.1016/j.jbc.2021.101074

**Published:** 2021-08-12

**Authors:** Dan Zhang, Kiera Robinson, Leonide Saad, Ilyas Washington

**Affiliations:** 1 Columbia University Medical Center, New York, New York, USA; 2 biOOrg3.14, Buffalo, Wyoming, USA

**Keywords:** C20D3-vitamin A, dark adaptation, retinal degeneration, vitamin A dimers, A2E AMD, A2E, *N*-retinylidene-*N*-retinylethanolamine, AF, autofluorescence, AMD, age-related macular degeneration, BM, Bruch's membrane, DA, dark adaptation, ERG, electroretinography, ICR, Institute of Cancer Research, qVAB, quantifiable VAB, RPE, retinal pigment epithelium, UPLC, ultraperformance liquid chromatography, VAB, vitamin A byproduct

## Abstract

Impaired dark adaptation (DA), a defect in the ability to adjust to dimly lit settings, is a universal hallmark of aging. However, the mechanisms responsible for impaired DA are poorly understood. Vitamin A byproducts, such as vitamin A dimers, are small molecules that form in the retina during the vitamin A cycle. We show that later in life, in the human eye, these byproducts reach levels commensurate with those of vitamin A. In mice, selectively inhibiting the formation of these byproducts, with the investigational drug C20D_3_-vitamin A, results in faster DA. In contrast, acutely increasing these ocular byproducts through exogenous delivery leads to slower DA, with otherwise preserved retinal function and morphology. Our findings reveal that vitamin A cycle byproducts alone are sufficient to cause delays in DA and suggest that they may contribute to universal age-related DA impairment. Our data further indicate that the age-related decline in DA may be tractable to pharmacological intervention by C20D_3_-vitamin A.

The vertebrate eye operates at a range of light intensities by adapting its sensitivity to different illumination levels. With age, the ability to adapt to variations in light intensity diminishes, leading to transient visual incapacitation or blindness when proceeding from brighter to darker settings. Impaired dark adaptation (DA) affects the ability to drive, navigate around objects while walking, and control balance and locomotion (1), increasing the risk of injuries from accidental falls (2).

Age-related declines in DA can occur in the absence of overt ocular pathology and are considered an inevitable consequence of aging (3). While senescent changes, such as increased lens rigidity, reduced pupillary response, and clouding of the ocular media, may account for age-associated deteriorations in focusing ability, light sensitivity, color perception, resistance to glare, or contrast sensitivity, the mechanisms responsible for declines in DA are less understood (3, 4). Furthermore, there are little data to suggest that age-related declines in DA can be prevented. Although delayed DA is considered a risk factor for the development of age-related macular degeneration (AMD) (5, 6), its role in the pathogenesis of AMD is not understood.

During the vitamin A cycle, a portion of retinaldehyde condensates with an amine, such as phosphatidylethanolamine, phosphatidylserine, and lysine resulting in the nonenzymatic dimerization of retinaldehyde (7, 8, 9). These dimers of retinaldehyde can be found in the retinal pigment epithelium (RPE) (9, 10) and Bruch's membrane (BM) (11), where they can be transformed into higher-order oligomers (12) and/or smaller oxidative catabolites (13, 14, 15, 16), molecules that, all together, can be termed vitamin A byproducts (VABs). We hypothesized that the VAB formation caused delayed DA and that preventing VAB formation would improve DA.

## Results

### Vitamin A cycle byproducts increase with age in the human retina

**Figure 1:**
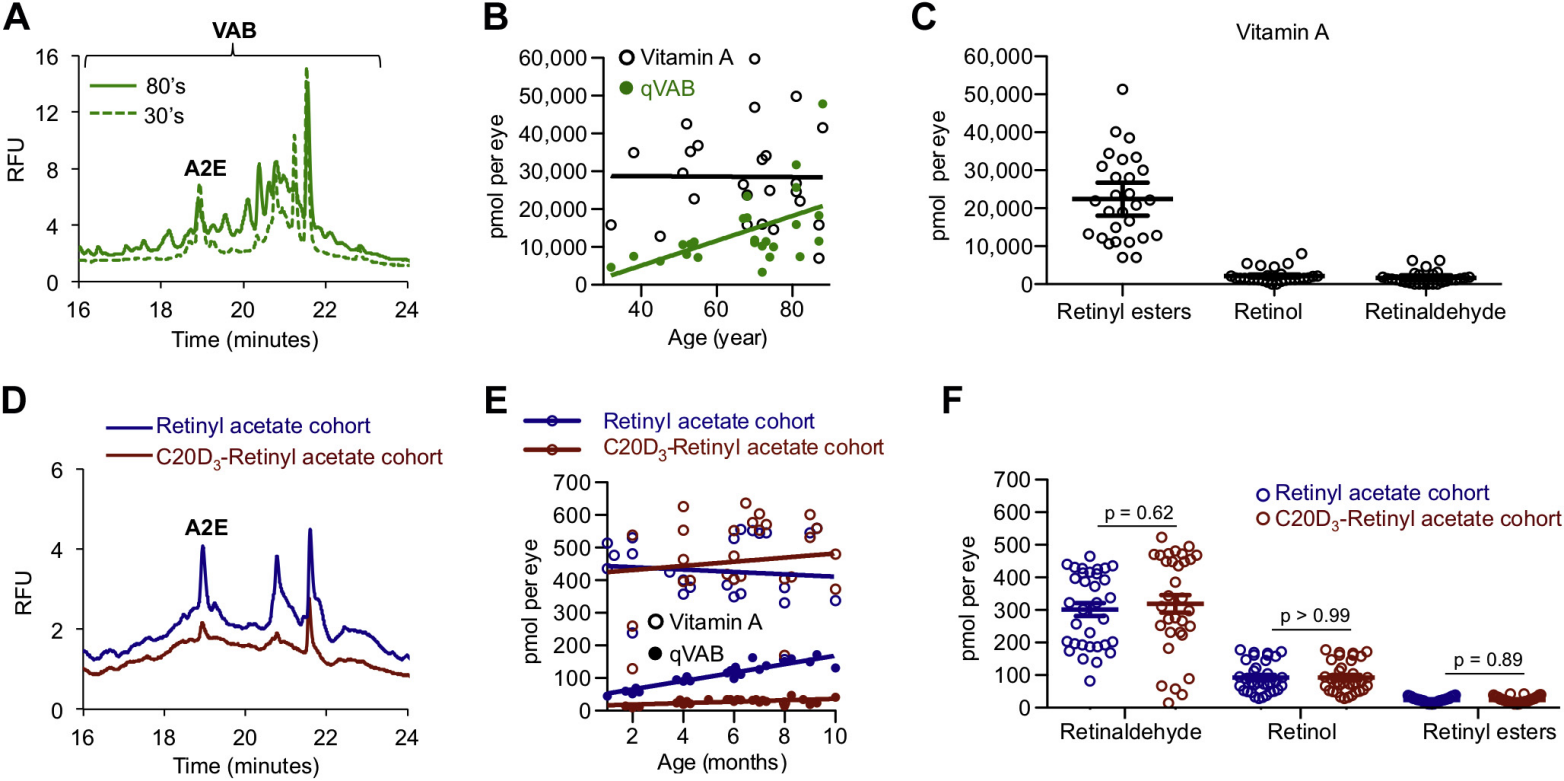
**Vitamin A cycle byproducts form with age; administration of C20D**_**3**_**-vitamin A retards byproduct formation.***A*, representative UPLC fluorescence signatures of extracts from human retina (neuroretina, RPE, and choroid). Excitation = 488 nm; emission = 650 nm. Each peak corresponds to a different VAB. 80s, n = 3 pooled eyes, 30s, n = 2 pooled eyes. *B*, vitamin A (retinyl palmitate, retinyl stearate, 11-*cis*-retinyl palmitate, and the *cis*, *trans* isomers of retinaldehyde and retinol) and qVAB (A2E, iso-A2E, and oxo-A2E) in the human retina. About 25 eyes from 25 donors (n = 25) were used in total. *C*, average amounts, with SEM, of vitamin A in 32- to 87-year-old, human retina (n = 25 eyes). Retinyl esters (mean ± SD): 22,404 ± 11,147 pmols per eye. Retinol: 2159 ± 1895 pmols per eye. Retinaldehyde: 1650 ± 1748 pmols per eye. *D*, representative UPLC traces of eye extracts from 9-month-old *Abca4*^−/−^/*Rdh8*^−/−^ mice administered a standard rodent diet containing vitamin A as retinyl acetate (*blue curve*) or C20D_3_-retinyl acetate (*red curve*) from weaning. *E*, vitamin A and qVAB in the eyes of *Abca4*^−/−^/*Rdh8*^−/−^ mice described in panel *D*. Each point represents between five and 10 pooled eyes. For the retinyl acetate cohort, 125 eyes were used (n = 125); for the C20D_3_-retinyl acetate cohort, n = 115. qVAB increased at a rate (with 95% confidence interval) of 13 (11–15) pmol per eye per month in the retinyl acetate cohort and 2 (0.7–4) pmol per eye per month for the C20D_3_-retinyl acetate cohort; an 83% decrease in the rate of qVAB accumulation, *p* < 0.0001, two-sided *F* test. *F*, average amounts (with SEM) of vitamin A in the eyes of the *Abca4*^−/−^/*Rdh8*^−/−^ mice described in panels *D* and *E*, from 3 to 10 months of age. A2E, *N*-retinylidene-*N*-retinylethanolamine; qVAB, quantifiable VAB; RFU, relative fluorescence unit; RPE, retinal pigment epithelium; UPLC, ultraperformance liquid chromatography; VAB, vitamin A byproduct.

VABs are implicated in the pathogenesis of several retinal disorders (7, 8, 9, 10, 11, 12, 13, 14, 15, 16). Age-related increases in fundus autofluorescence (AF)—as observed upon 488-nm excitation and 500- to 680-nm emission—are considered a biomarker for retinal aging (17) and thought to reflect the amount of VAB. Using ultraperformance liquid chromatography (UPLC), we measured AF signatures of retina extracts (neuroretina, RPE, and choroid) upon 488-nm excitation and 650-nm emission. This method excludes emissions from vitamin A (and its congeners), which does not emit light at these wavelengths. We detected 19 AF chemical species in human eyes in the third decade of life, which increased to 32 species by the eighth decade of life (Fig. 1*A*), consistent with an age-related increase in the number of VAB. To quantify the VAB, we measured the amounts of three well-characterized byproducts: *N*-retinylidene-*N*-retinylethanolamine (A2E), its geometric isomer, iso-A2E, and its major oxidative metabolite, oxo-A2E (Fig. S1, *A* and *B*), altogether denoted as quantifiable VAB (qVAB). We found that qVAB accrued linearly at a rate of approximately 64 pmols per year of life (Fig. 1*B*).

Since the kinetics of DA depend on the efficiency of the vitamin A cycle, which itself necessarily relies on concentrations of vitamin A congeners (18), we measured the concentrations of ocular vitamin A congeners (Fig. 1, *B* and *C* and Fig. S1, *C*–*E*). We found that the amounts of retinyl esters, retinol, and retinaldehyde were independent of age (Fig. S2), suggesting that age-related declines in DA could occur in the absence of local vitamin A deficiencies. Vitamin A existed predominantly as retinyl esters followed by approximately equal amounts of retinol and retinaldehyde. There was a moderate positive correlation between the amount of A2E and retinol (Pearson correlation coefficient: 0.57, two-tailed *p* value: 0.003). The amount of qVAB exceeded that of retinol and retinaldehyde at all examined ages and on average equaled and in some cases surpassed the aggregate concentration of vitamin A congeners after approximately 70 years of age. The relatively large amount of VAB suggested that the byproducts played a role in the age-related deterioration of visual performance.

### VAB and the 650-nm fluorescent species are derived from hydrogen abstraction from C20 of vitamin A

To elucidate the consequence of VAB formation on DA, we used mice with abolished Abca4 and Rdh8 activity (19) (*Abca4*^−/−^/*Rdh8*^−/−^). The *Abca4* and *Rdh8* genes encode for proteins in the vitamin A cycle that transport and reduce retinaldehyde, respectively. Their absence results in the increased formation of VAB (19). To modulate VAB formation, we provided mice from weaning with matched diets where vitamin A was supplied as either retinyl acetate or as C20D_3_-retinyl acetate. Deuterium enrichment at carbon number 20 of vitamin A results in a kinetic isotope effect that slows the dimerization of retinaldehyde without modifying the vitamin A cycle (20). In 9-month old *Abca4*^−/−^/*Rdh8*^−/−^ mice given retinyl acetate, over twenty-five 650-nm fluorescent species were present in the UPLC AF signatures, compared with 15 in the C20D_3_-retinyl acetate cohort (Fig. 1*D*). This decrease in the number and amount of 650-nm fluorescent species, upon administration of C20D_3_-retinyl acetate, further demonstrated that the fluorescent species were derived from vitamin A, and the rate-limiting step in their formation was hydrogen abstraction from C20 of vitamin A (20, 21, 22).

qVAB accumulated 83% more slowly with age in the C20D_3_-retinyl acetate cohort than the retinyl acetate cohort (Fig. 1*E*). Similar to humans, the amounts of retinol, retinaldehyde, and retinyl esters were independent of age and identical in both cohorts (Fig. 1*F* and 3*A*). In the mice, the most abundant vitamin A congener was retinaldehyde, followed by retinol and retinyl esters (Fig. 1*F*).

**Figure 2:**
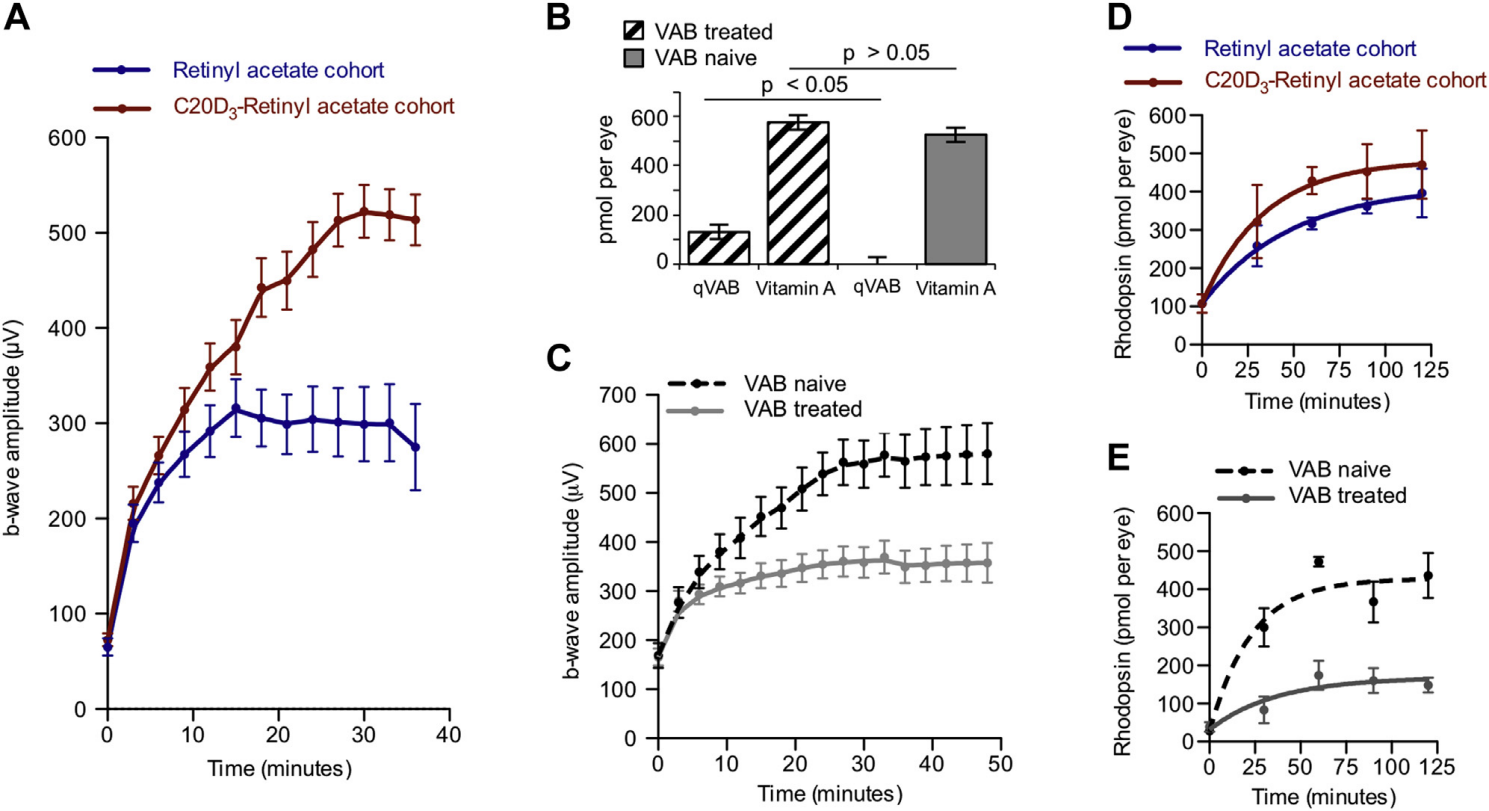
**Vitamin A cycle byproducts, delay dark adaptation.***A*, average (SEM) ERG b-wave recoveries following light exposure, for ∼5-month-old, *Abca4*^−/−^/*Rdh8*^−/−^ mice administered a diet containing retinyl acetate (n = 16 eyes, *blue curve*, recovered a mean and SEM of 300 ± 30 μV after 30 min, when fitted to an exponential curve) or C20D_3_-retinyl acetate (n = 24 eyes, *red curve*, recovered 561 ± 29 μV, after 30 min; a 87% increase, *p* = 0.0001, two-sided *F* test). *B*, average (SEM) amounts of ocular vitamin A and qVAB following a single intraocular injection of VAB (VAB-treated, n = 3) or sham (VAB-naive, n = 3) into wildtype mice. *C*, average with SEM, ERG b-wave recoveries following light exposure in VAB-naive (n = 13 eyes, *dashed curve*, recovered 600 ± 39 [SEM] μV) and VAB-treated wildtype mice (n = 18 eyes, *solid curve*, recovered 355 ± 9 μV, *p* = 0.003, two-sided *F* test). *D* and *E*, rhodopsin recoveries in mice described in panels *A* through *C* fitted to an exponential curve. *D*, retinyl acetate cohort, n = 10 eyes, rate constant with SEM = 0.020 ± 0.009 min^−1^. C20D_3_-retinyl acetate cohort, n = 10 eyes, rate constant with SEM = 0.029 ± 0.015 min^−1^, *p* = 0.048, two-sided *F* test. *E*, VAB-naive, n = 30 eyes, rate constant with SEM = 0.044 ± 0.018 min^−1^. VAB-treated, n = 28 eyes, rate constant with SEM = 0.027 ± 0.024 min^−1^, *p* = 0.0001, two-sided *F* test. ERG, electroretinography; qVAB, quantifiable VAB; VAB, vitamin A byproduct.

### VAB causes delayed DA

**Figure 3:**
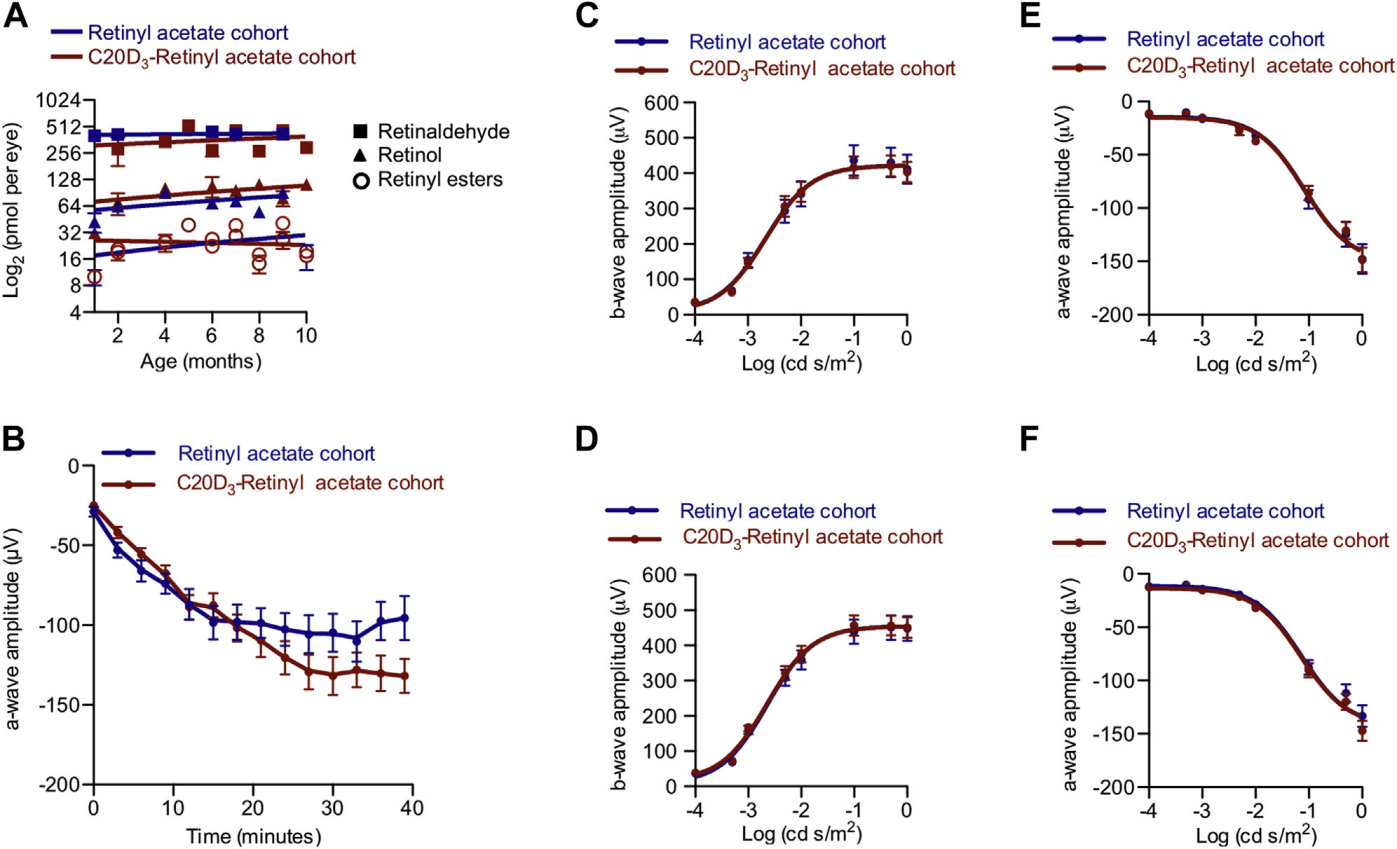
**Visual cycle byproducts delay dark adaptation, despite otherwise normal retina normal electrophysiological function.***A*, amounts with SEM of retinyl esters (retinyl palmitate, retinyl stearate, and 11-*cis*-retinyl palmitate), retinol (*cis* and *trans* isomers), and retinaldehyde (*cis* and *trans* isomers), in *Abca4*^−/−^/*Rdh8*^−/−^ mice administered, from weaning, a standard rodent diet containing retinyl acetate (n = 167, *blue curve*) or C20D_3_-retinyl acetate (n = 167, *red curve*). All slopes did not significantly deviate from zero, as determined by *p* values >0.5 from *F* tests. *B*, average with SEM of ERG a-wave recoveries following light exposure, in ≈5-month-old, *Abca4*^−/−^/*Rdh8*^−/−^ mice administered a diet containing retinyl acetate (n = 28 eyes, *blue curve*, recovering a mean and SEM of 104 ± 5 μV) or C20D_3_-retinyl acetate (n = 16 eyes, *red curve*, recovering 153 ± 14 μV, *p* = 0.0299, two-sided *F* test). *C*–*F*, ERG dose response curves (average with SEM) for the cohorts of dark-adapted *Abca4*^−/−^/*Rdh8*^−/−^ mice described in panel *B* at 5 months (*C* and *D*, retinyl acetate, n = 15 eyes; C20D_3_-retinyl acetate, n = 23 eyes) and 9 months of age (*E* and *F*, retinyl acetate, n = 18 eyes; C20D_3_-retinyl acetate, n = 25 eyes). ERG, electroretinography.

The ability to dark adapt can be quantified by measuring the time it takes to perceive flashes of light of varying intensities or by the time it takes the electroretinography (ERG) b-wave to recover (23) after being placed in the dark following bright light exposure. By the second half of life, it takes approximately 50% longer for one to discern relatively dim test flashes compared with when in one's 20s (24). We thus measured b-wave recoveries in the aforementioned cohorts of *Abca4*^−/−^/*Rdh8*^−/−^ mice. Because acclimating to mesopic luminance plays a more significant role in navigating environments than scotopic luminance, and because both rod-mediated and cone-mediated (25) visions undergo progressive slowing of DA with age, we used mesopic test flashes. With this protocol, *Abca4*^−/−^/*Rdh8*^−/−^ animals administered C20D_3_-retinyl acetate recovered 87% higher b-wave amplitudes after 30 min of DA, compared with animals administered retinyl acetate (Fig. 2*A*). For reference, a 25%, 50%, or 80% decrease in recovered b-wave amplitudes, measured 30 min after being placed in the dark, induced by administration of the RPE65 inhibitor emixustat, correlated with complaints of difficulty seeing at night in 25%, 50%, and 80% of subjects, respectively (26). Thus, the observed 87% higher b-wave amplitudes would be clinically relevant. ERG a-wave recoveries were improved by 46% in the C20D_3_-retinyl acetate cohort (Fig. 3*B*). At the same time, dark-adapted maximum a-wave and b-wave amplitudes (Fig. 3, *C*–*F*) and the integrity and thickness of retinal layers (Fig. S3, *A–E*) were statistically indistinguishable between the cohorts. Though, there was marginally more total rhodopsin in the C20D_3_-retinyl acetate cohort (Fig. S3*F*). These data indicate that, as in humans, declines in DA could appear absent of otherwise pathological changes in retinal electrophysiological function or morphology.

To determine if the slowed ERG recoveries were due to VAB, or independent age-related changes, such as changes in pupillary reflex or changes in the BM (24), we injected a bolus of VAB intravitreally into 3-month-old wildtype mice and shortly thereafter measured a-wave and b-wave recoveries. VAB treatment promptly increased the amount of qVAB to ∼20% of total vitamin A, a level relatively similar to that found in 5-month-old *Abca4*^−/−^/*Rdh8*^−/−^ animals (Fig. 2*B*). Increased VAB resulted in a 41% and 37% depression of b-wave (Fig. 2*C*) and a-wave (Fig. S4*A*) amplitudes after 30 min of DA, respectively, compared with sham-injected and noninjected control animals. These decreased ERG recoveries were apparent despite a lack of significant changes in maximum dark-adapted ERG a-wave and b-wave amplitudes (Fig. S4, *B* and *C*), light histology (Fig. S4, *D*–*G*), or total rhodopsin (Fig. S4*H*) between the two cohorts. Similar results were obtained when using Syrian hamsters (*Mesocricetus auratus*) in place of mice (Fig. S5). Taken together, these data suggest that the association between delayed a-wave and b-wave recoveries and an increase in ocular VAB, relative to total vitamin A, were neither specific to the genetic makeup of the mice, namely the *Abca4*^−/−^/*Rdh8*^−/−^ mutations nor dependent on changes in the BM, or pupillary reflex, which would presumably occur over longer time frames after VAB administration.

Delayed DA can result from changes in the phototransduction cascade or rhodopsin regeneration (24, 27). We found that rhodopsin regeneration was 31% and 39% slower in the *Abca4*^−/−^*/Rdh8*^−/−^ animals administered retinyl acetate and wildtype animals treated with VAB, respectively, compared with *Abca4*^−/−^/*Rdh8*^−/−^ animals administered with C20D_3_-retinyl acetate or VAB-naive animals (Fig. 2, *D* and *E*). This indicated that the observed delays in a-wave and b-wave recoveries could be attributed to delayed rhodopsin regeneration, consistent with delays in rhodopsin regeneration observed in humans with delayed DA (24).

## Discussion

### Delayed DA leads to retinal degeneration

The inability to regenerate rhodopsin results in prolonged activation of the visual cascade to the detriment of the retina termed constitutive signaling (28, 29, 30). In the prolonged light-adapted state, oxygen consumption is lower, leading to higher oxygen concentrations in the retina (31, 32) and thus increased oxidative damage (33, 34, 35, 36). In contrast, when dark adapted, the retina's oxygen consumption is maximal, which reduces the amount of oxygen available to cause oxidative damage. Indeed, symbiotic theory proposes that oxygen consumption *via* mitochondrial respiration evolved to protect against increasing environmental oxygen. Furthermore, delays in DA alter the concentration of retinoic acid (37), a molecule, which regulates a variety of functions in the retina.

Constitutive signaling, increased retinal oxygen, and altered retinoic acid concentrations are responsible, in part or in combinations, for retinal degenerations resulting from vitamin A deficiency, constant light exposure, Leber congenital amaurosis, congenital night blindness, RPB4 deficiency, and retinitis pigmentosa. The aforementioned mechanisms also explain why keeping animals in the dark (dark adapted) mitigates several forms of retinal degeneration (38, 39, 40). Similarly, retinal degeneration would be expected from chronic transient delays in DA caused by VAB. Indeed, delayed DA is often observed years before the onset of AMD (41, 42).

### VAB may impede DA by several mechanisms

The structures of a majority of VAB remain to be determined. As such, we used qVAB as a surrogate for the amount of VAB, thereby underestimating the amount of total VAB. The relatively high quantities of qVAB relative to vitamin A in the human eye open the possibility that VAB, because of structural similarities to vitamin A, may compete with vitamin A cycle proteins. Indeed, the VAB A2E binds to RPE65 (43) and the retinoic acid receptor (44), and oxidative VAB catabolites (13, 14, 15, 16), such as β-ionone, may inhibit opsin (45), which could all impede the vitamin A cycle and thus slow DA. Delayed DA may also be caused by general VAB-induced toxicity or crowding of the RPE.

Genetic defects, which accelerate the accumulation of VAB, such as Stargardt and Sorsby's fundus dystrophies, can lead to delayed DA in young individuals—independent of the extent of pathology (46), similar to what was observed here. Observations that not all people with elevated amounts of VAB—because of aging or the aforementioned inherited retinal conditions—display DA defects (47), indicate that phenotypic heterogeneity, subjective vision, environmental factors, gene expression patterns, and genetic polymorphisms all likely influence the degree to which VAB disrupts DA for any particular individual.

### Preventing VAB formation to improve DA

By replacing dietary vitamin A with C20D_3_-retinyl acetate, the rate of formation of VAB was dramatically lowered, resulting in faster DA. This work does not suggest that DA defects are only caused by VAB. Rather, this work demonstrates that the age-related accumulation of VAB is sufficient alone to drive the worsening of DA. In the context of data demonstrating that declines in DA can be perceived and measured clinically within a few years (42, 48, 49), we suggest that C20D3-retinyl acetate can be used as a clinically amiable tool to determine the extent to which VAB participates in this universal deterioration of visual performance.

## Experimental procedures

### Interventions

Animal studies were approved by Columbia University's Institutional Animal Care and Use Committee. The *Abca4*^−/−^/*Rdh8*^−/−^ mice were obtained from Case Western Reserve University (19). The lighting in the vivarium was ≥0.05 μmol s^−1^ m^−2^ at the cage floor and ≈3 μmol s^−1^ m^−2^ in the room. The *Abca4*^−/−^/*Rdh8*^−/−^ animals were fed a diet containing 20,000 i.u. of vitamin A, as either retinyl acetate (Sigma–Aldrich) or C20D_3_-retinyl acetate (provided by Alkeus Pharmaceuticals) per kilogram of diet. Diets were formulated by Land O'Lakes, Inc as reported in the literature (21). Animals were divided into the two cohorts at random. Each cohort contained approximately half males and half females. No blinding was performed. For the wildtype experiments, 3-month-old, male, Institute of Cancer Research (ICR) mice and 3-month-old, half male and half female, hamsters were used, both purchased from Charles River. For intraocular delivery of the VAB A2E, pupils were dilated and analgesized by applying a drop of tetracaine (0.5%), tropicamide (1%), and phenylephrine hydrochloride (2.5%), animals were anesthetized with isoflurane, and 5 μl of a solution of 0.5 mg of A2E per milliliter of PBS, containing 0.5% dimethyl sulfoxide was injected, intraocularly, according to the literature procedures (50). ERG, histology, rhodopsin regeneration and quantification, and qVAB quantification were performed 3 to 5 days postdelivery of VAB. Injection of the vehicle alone (5 μl of PBS with 0.5% dimethyl sulfoxide, sham-injected cohort) did not significantly alter the kinetics of the recovery of the ERG a and b waves. Thus, data for the sham-injected and noninjected animals were combined and denoted as “VAB-naive” animals. Animals were divided into the two cohorts (VAB-naive and VAB-treated cohorts) at random. No blinding was performed.

### Retinoid isolation and quantification

For UPLC quantification, the retinyl palmitate, all-*trans* retinaldehyde, all-*trans*-retinol reference standards were purchased from Sigma–Aldrich. A2E was purified by flash silica gel chromatography, eluting with ethyl acetate:isopropyl alcohol:water (4:2:1) with 0.1% NH_4_OH. The counter ion was changed to trifluoromethanesulfonate to give a salt with a molecular weight of 742. The purity of the A2E standard was determined to be 98% by quantitative ^1^H-NMR, using 3,5-dinitrobenzoic acid as internal standard (Fig. S6). The calculated extinction coefficient in isopropanol was 35,490 at 440 nm.

Eyes were extracted according to the literature procedure (21). However, we used 300 μl of absolute ethanol in place of butanol as an extracting solvent, five to ten eyes for mice, and one eye for hamsters. About 30 μl of the extract was used for UPLC. For UPLC, a 50 × 2.1 mm, Kinetex 2.6 μm, C18, 100 Å column (Phenomenex; part number: 00B-4462-AN) was used. The flow rate was 1.5 ml/min, and compounds were eluted with solvent A for 1 min, followed by a linear gradient to 100% solvent B over 5 min, followed by 100% solvent B for an additional 2 min. A2E was detected at 445 nm, retinaldehyde at 380 nm, and retinol and retinyl esters with 325 nm. For quantification of iso-A2E and oxo-A2E, we used UPLC retention times and peak areas measured by a photodiode array detector at 445 ± 5 nm, using the same extinction coefficient as A2E.

For estimation of recoveries, known amounts of A2E, retinyl palmitate, all-*trans*-retinol, and all-*trans* retinaldehyde, in ethanol, were added to five dehydrated eyes from 3-month-old male ICR mice (Taconic). These mice carry the Pde6b^rd1^ mutation and thus lack ocular retinoids. The resulting mixture was extracted and analyzed by UPLC, as described for the study eyes. The lower limit of detection for A2E was 2 pmol per eye when using five eyes (as most commonly done). The lower limit of quantification for A2E was 5 pmol per eye when using five eyes.

Recovery was 92 ± 5% (SD) for A2E, 97 ± 7% for retinyl palmitate, 99 ± 4% for all-*trans* retinaldehyde, and 94 ± 10% for all-*trans*-retinol, when known amounts of the respective analytes were spiked into samples not containing eyes. Male ICR mice eyes from Taconic contained undetectable amounts of A2E and retinaldehyde, 9 ± 5 pmol per eye retinol, and 13 ± 8 pmol per eye retinyl esters (n = 15). Accordingly, when known amounts of the analytes were spiked into samples containing five ICR mice eyes, we were able to recover 94 ± 8% of A2E, 89 ± 11% of retinyl palmitate, 91 ± 5% of all-*trans* retinaldehyde, and 85 ± 5% of all-*trans*-retinol.

UPLC fluorescence profiles were obtained by pooling eye extracts, concentrating them, and injecting 77 μl in the UPLC system. A 2.1 × 150 mm, Brownlee SPP 2.7 μm column was used (part number N9308405; PerkinElmer). The flow rate was 0.5 ml/min, and compounds were eluted with solvent A for 5 min, followed by a linear gradient to 100% solvent B over 15 min, followed by 100% solvent B for an additional 8 min at a flow rate of 1 ml/min.

### ERG

ERG a-wave and b-wave amplitudes were recorded and analyzed following the literature procedures (51, 52). For quantification of DA kinetics, the same ERG system was used, and animals were prepared as described. For bleaching, *Abca4*^−/−^/*Rdh8*^−/−^ mice and hamsters were illuminated, whereas in the ganzfeld apparatus, with a 530-nm LED light of 300 cd/m^2^ (scotopic), for 5 min. The ICR mice were illuminated with the same light, but at 100 cd/m^2^ (scotopic), for 5 min. Xenon probe flashes of 0.5 cd s/m^2^ (scotopic) were presented in triplicate every 3 min. To calculate recovered ERG, the average a-wave and b-wave amplitudes in microvolt for the three probe flashes were plotted *versus* time and fitted to a one-phase exponential equation. Each eye was used as a statistical unit. The study was powered to detect at least a 50% treatment effect in b-wave recoveries.

### Rhodopsin regeneration and quantification

For bleaching, pupils were dilated and analgesized by applying a drop of a solution containing tetracaine (0.5%), tropicamide (1%), and phenylephrine hydrochloride (2.5%). Five animals were placed into a cage with an open top and exposed to fluorescent light of 50 μmol s^−1^ m^−2^ measured at the cage floor for 30 min. For DA, the light was removed, and the animals were placed in the dark. Rhodopsin isolation was conducted under 660-nm light. Each eye was put into a 1.5-ml centrifuge tube and sheered with scissors. We added 0.5 g of 0.5-mm zirconium oxide beads, 500 μl of buffer (0.1% 2-mercaptoethanol, 0.05 M hydroxylamine, 2% lauryldodecylamine oxide in PBS), and homogenized the eyes with a bead mill homogenizer (BBY24M Bullet Blender STORM; Next Advance, Inc, setting seven for 5 min). The homogenates were centrifuged at 14,000 rpm, at 4 °C, for 25 min, and 200 μl was placed into a well of a 96-well plate. The amount of rhodopsin was calculated by measuring the decrease in absorbance at 500 nm (using a reference at 700 nm) after light exposure, using an extinction coefficient of 42,700 l mol^−1^ cm^−1^. To calculate rate constants, the amount of rhodopsin was plotted *versus* time and fitted to a one-phase exponential equation. Each eye was used as a statistical unit.

### Histology

Whole eyes were enucleated, fixed in Excalibur's fixative (Excalibur Pathology, Inc), embedded in paraffin, sectioned randomly, and stained with hematoxylin/eosin, following standard procedures. A minimum of ten sections were made for each eye. For each eye, three of the ten sections were chosen at random and imaged at 40×. Measurements of retinal thickness were made from both sides at a distance between 440 and 720 μm from the optic nerve head. For each slice, six measurements, three on each side of the optic nerve head, were made for each of six retinal layers. To determine the thickness of each retinal layer, for each eye, 18 measurements (six measurements × three slices per eye) were averaged. Each eye was used as a statistical unit.

### Statistics

All statistical analyses were performed with GraphPad Prism (GraphPad Software, Inc). Values are shown as mean ± SEM or SD. A *p* value of less than 0.05 was considered significant as calculated by a two-sided unpaired *t* test or *F* test, where appropriate.

## Data availability

All remaining data are contained within the article.

## Supporting information

This article contains supporting information.

## Conflict of interest

I. W. is an inventor on patents disclosing methods to prevent retinal degeneration. All other authors declare that they have no conflicts of interest with the contents of this article.
