# Supplementary material for: Vitamin A cycle byproducts impede dark adaptation
Source: J Biol Chem. 2021 Aug 12;297(3):101074. doi: 10.1016/j.jbc.2021.101074 (PMC8427233; doi:10.1016/j.jbc.2021.101074)
Supplement: Figures S1–S6 [file mmc1.docx]

**Vitamin A cycle byproducts impede dark adaptation**

Dan Zhang^1^, Kiera Robinson^1^, and Leonide Saad^1^, Ilyas Washington^1,2.*^

^1^Columbia University Medical Center, New York, NY

^2^biOOrg3.14, Buffalo, WY, USA

**Supporting Information**

**
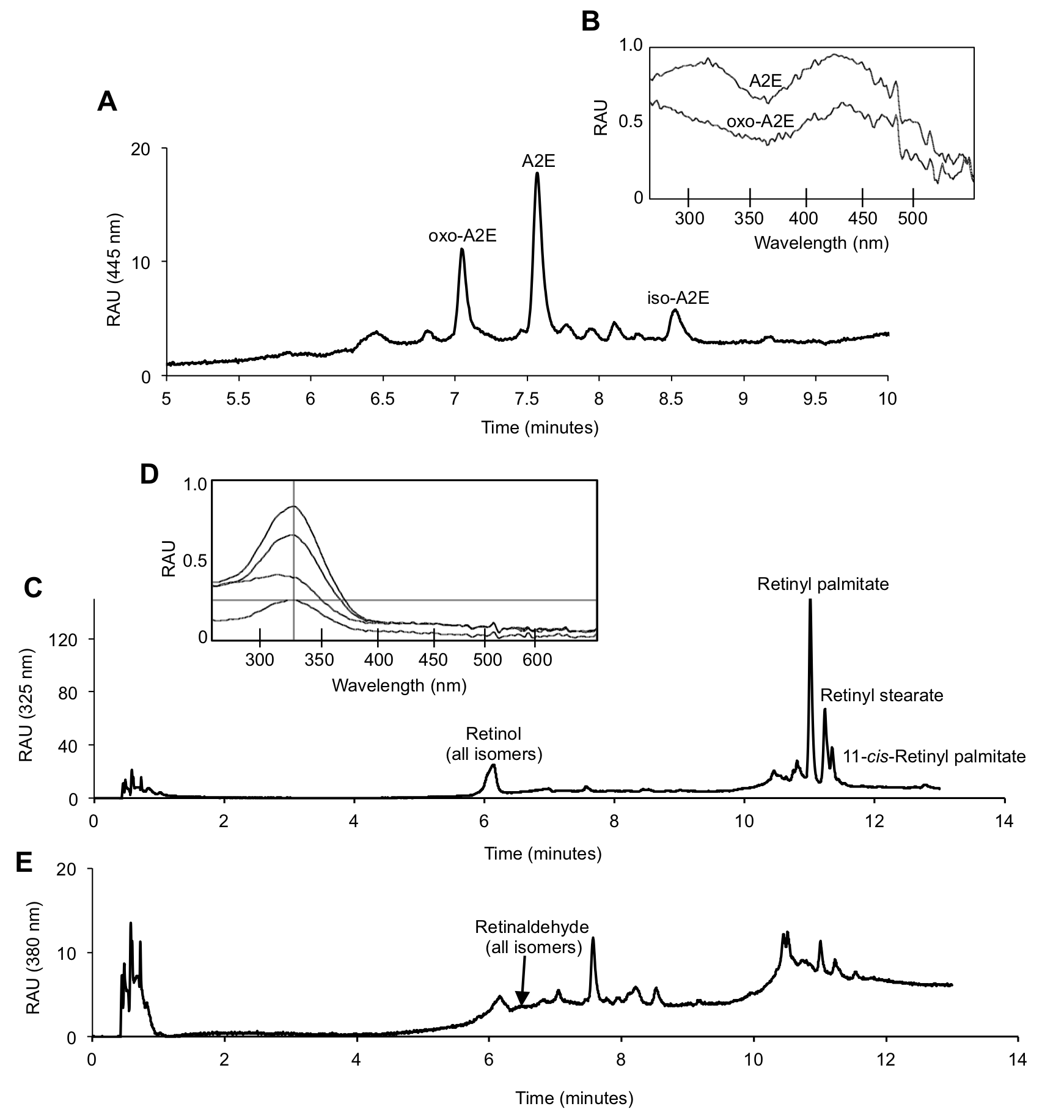
**

**Supporting Information Figure 1. qVAB and retinoid quantification by UPLC**

(**A**) Representative UPLC curve of an extract from an 87-year-old human retina (neuroretina, RPE and choroid) measured at 445 nm. Peaks representing A2E, iso-A2E and oxo-A2E (“qVAB”) are shown.

(**B**) Representative UV-Vis spectra of A2E and oxo-A2E from panel **a**.

(**C**) Representative UPLC curve measured at 325 nm, of an extract from an 87-year-old human retina (neuroretina, RPE and choroid). Peaks representing retinyl esters (retinyl palmitate, retinyl stearate, and 11-*cis*-retinyl palmitate) and retinol (*cis* and *trans* isomers) are shown.

(**D**) Representative UV-Vis spectra of peaks in panel **c**, representing retinyl esters and retinol, all peaking at approximately λ_max_ = 325-nm.

(**E**) Representative UPLC curve, measured at 380 nm, of an extract from an 87-year-old human retina (neuroretina, RPE and choroid). The location of the peak representing retinaldehyde (*cis* and *trans* isomers) is shown.


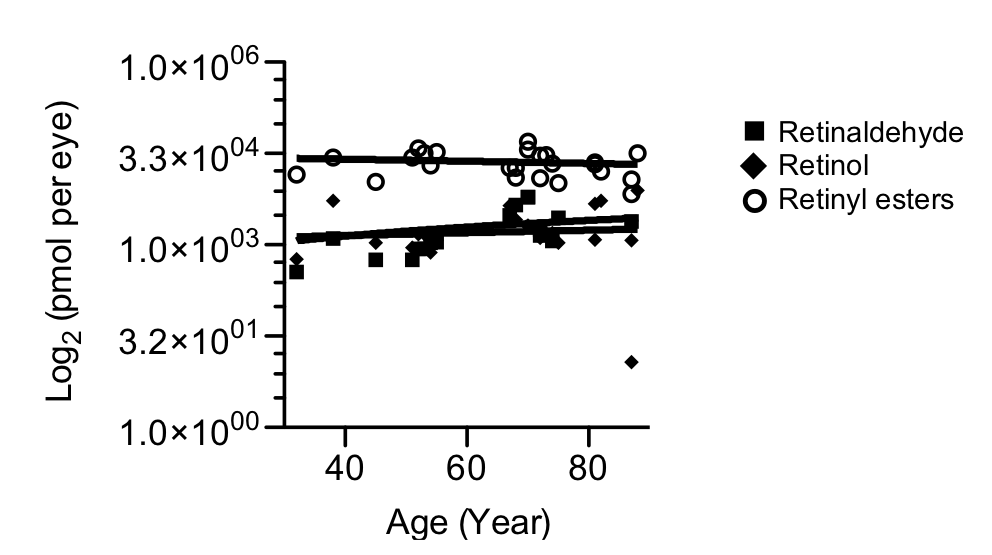


**Supporting Information Figure 2. Vitamin A congeners with age in the human retina**

Amounts of retinyl esters (retinyl palmitate, retinyl stearate, and 11-*cis*-retinyl palmitate), retinol (*cis* and *trans* isomers) and retinaldehyde (*cis* and *trans* isomers) in the human retina, with age. N = 25 eyes from 25 donors were used in total. All slopes did not significantly deviate from zero, as determined by P-values > 0.25 from F-tests performed for each vitamin A congener, indicating that there was no effect of age on the amounts of these vitamin A congeners.


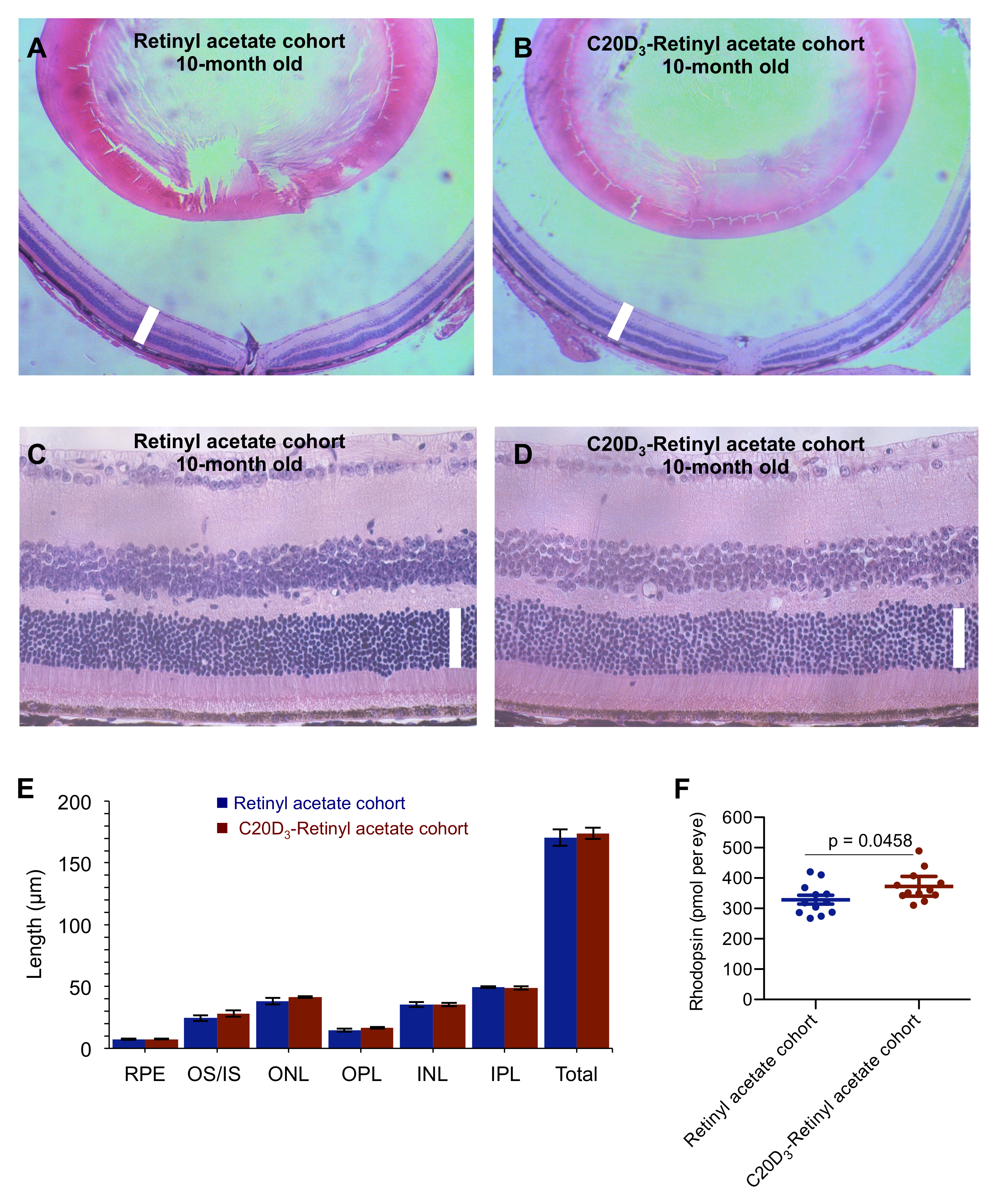


**Supporting Information Figure 3. Delayed ERG recoveries, despite otherwise normal morphology.**

(**A-D**) Representative retinal cross sections of 10-month old, *Abca4*^-/-^/*Rdh8*^-/-^ mice administered a diet containing retinyl acetate or C20D_3_-retinyl acetate. No pathological changes were observable. White scale bar is 170 μm long in panels A and B and 40 μm lung in panels C and D.

(**E**) Quantification of retinal layer thickness, 200 μm from either side the optic nerve head, in mice described in panels **A** through **D**. Retinyl acetate (n = 4), C20D_3_-retinyl acetate cohort (n = 4).

(**F**) Total dark-adapted rhodopsin in mice described in panels **A** through **D**, 9 to 10 months of age. Each point represents one eye. Mean and SEM are shown. There was 13% more dark-adapted rhodopsin in the C20D_3_-retinyl acetate cohort compared to the retinyl acetate cohort (mean and SEM: 329 ± 15 pmol/eye, n = 12 vs. 373 ± 15 pmol/eye, n = 12, P < 0.05, two-sided, unpaired, T-test).


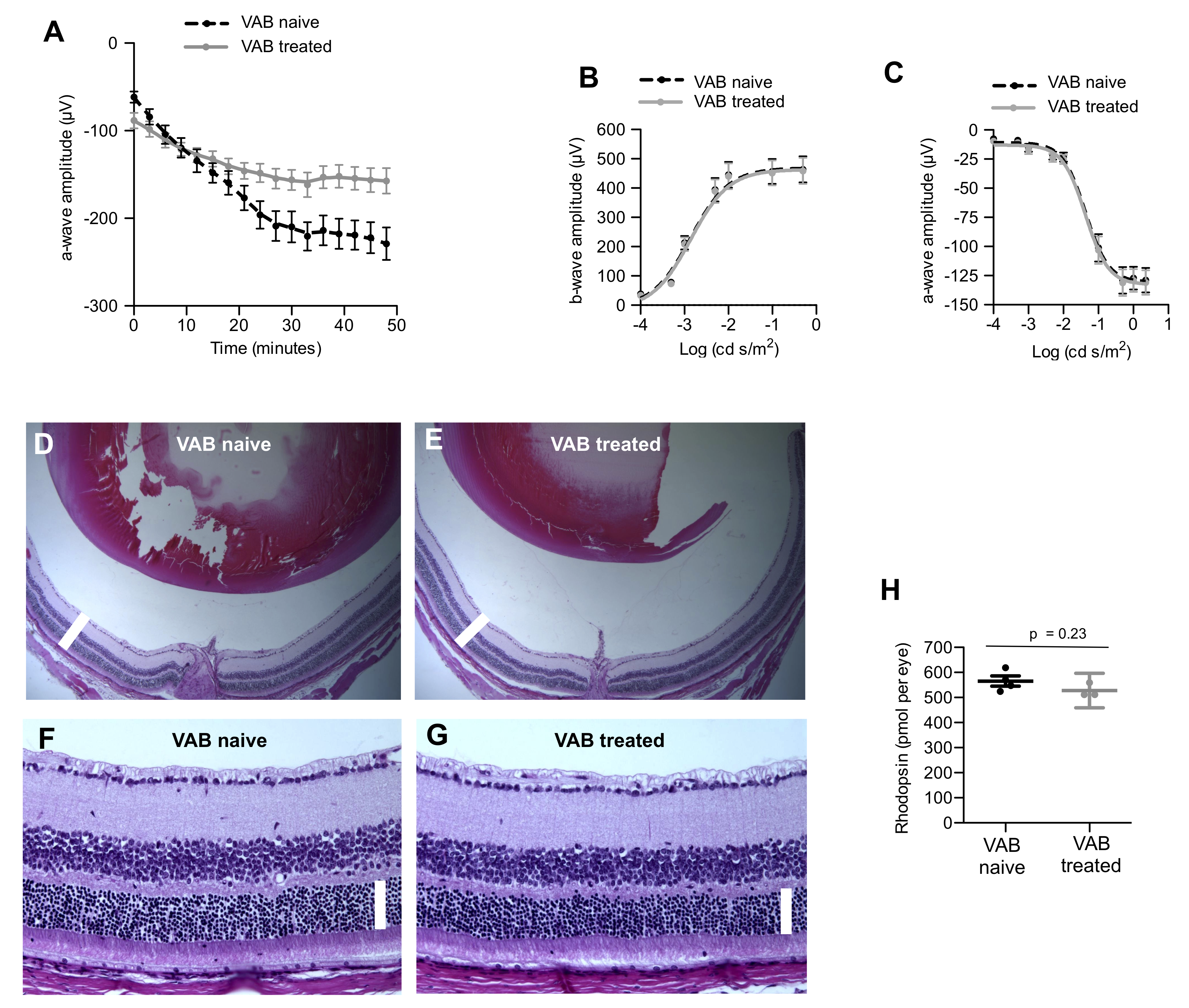


**Supporting Information Figure 4. Delivery of visual cycle byproducts delay dark adaptation in mice before altering morphology or electrophysiological function.**

(**A**) Average with SEM of ERG a-wave recoveries of wild-type, ICR mice treated with an intraocular injection of VAB (VAB-treated, n = 18 eyes, solid line, recovered -160 ± 7 μV) or sham (VAB-naive, n= 18 eyes, dashed line, recovered -253 ± 20 μV). For VAB-naive mice, a-waves recovered 47% higher compared to the VAB-treated mice after ≈ 30 min (P = 0.012, two-sided, F-test).

(**B and C**) ERG dose response curves (average with SEM) for dark adapted wild-type mice that are either VAB treatment naive (n = 18 eyes, dashed line) and VAB-treated (n = 18 eyes, solid line).

(**D-G**) Representative retinal cross sections of VAB-naive and VAB-treated animals. We observed no pathological changes induced by the delivery of VAB at the time of measuring ERG recoveries, when comparing five VAB treated to five VAB naive eyes. White scale bar is 170 μm long in panels D and E and 40 μm lung in panels F and G.

(**H**) Total dark-adapted rhodopsin in mice described in panel **a** through **G**. Each point represents one eye. Mean and SEM are shown, P value from a two-sided, unpaired, T-test.


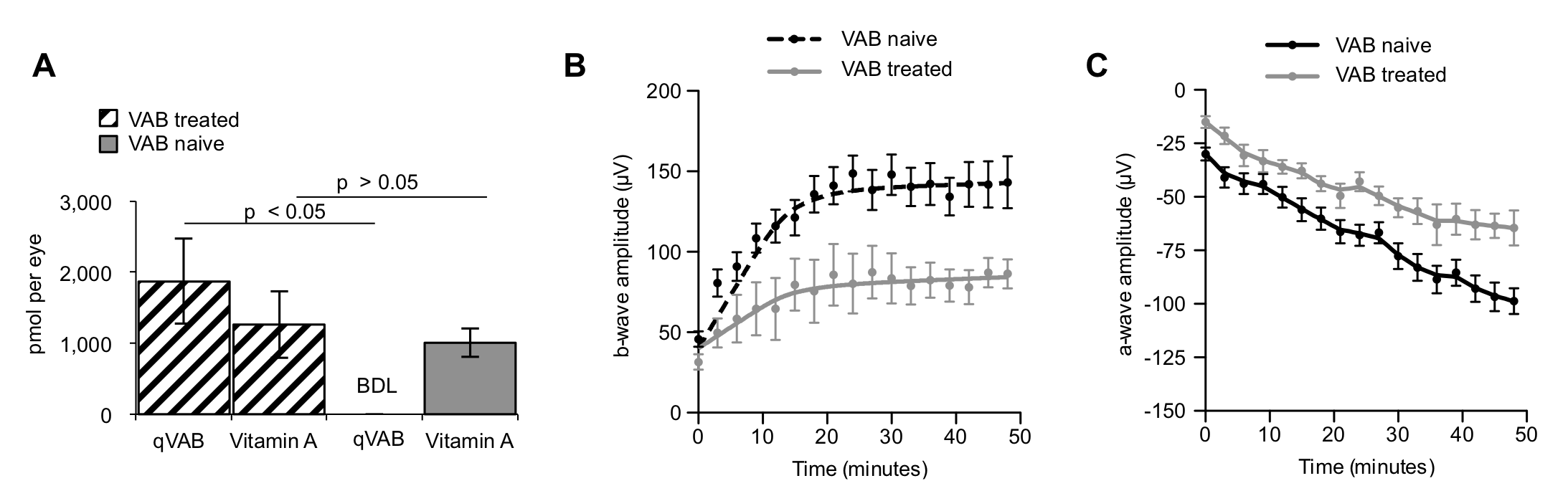


**Supporting Information Figure 5. Delivery of visual cycle byproducts delay dark adaptation in hamsters**

(**A**) Mean (SEM) amounts of ocular Vitamin A and qVAB following intraocular injection of VAB (VAB-treated, n = 7) or sham injection (VAB-naive, n = 7, P values from a two-sided, unpaired, T-test). BDL: Below detection limits.

(**B**) Average with SEM of ERG b-wave recoveries following light exposure in VAB-naive hamsters (n = 35 eyes, dashed line, recovered to 144 ± 5 μV) and VAB-treated hamsters (n = 6 eyes, solid line, recovered to 84 ± 5 μV, or a difference of 50%, P = 0.0001, two-sided F-test).

(**C**) Average with SEM of ERG b-wave recoveries following light exposure in VAB-naive (n = 34 eyes, dashed line, recovered to 99 ± 6 μV) and VAB-treated hamsters (n = 6 eyes, solid line, recovered to 64 ± 8 μV).

**
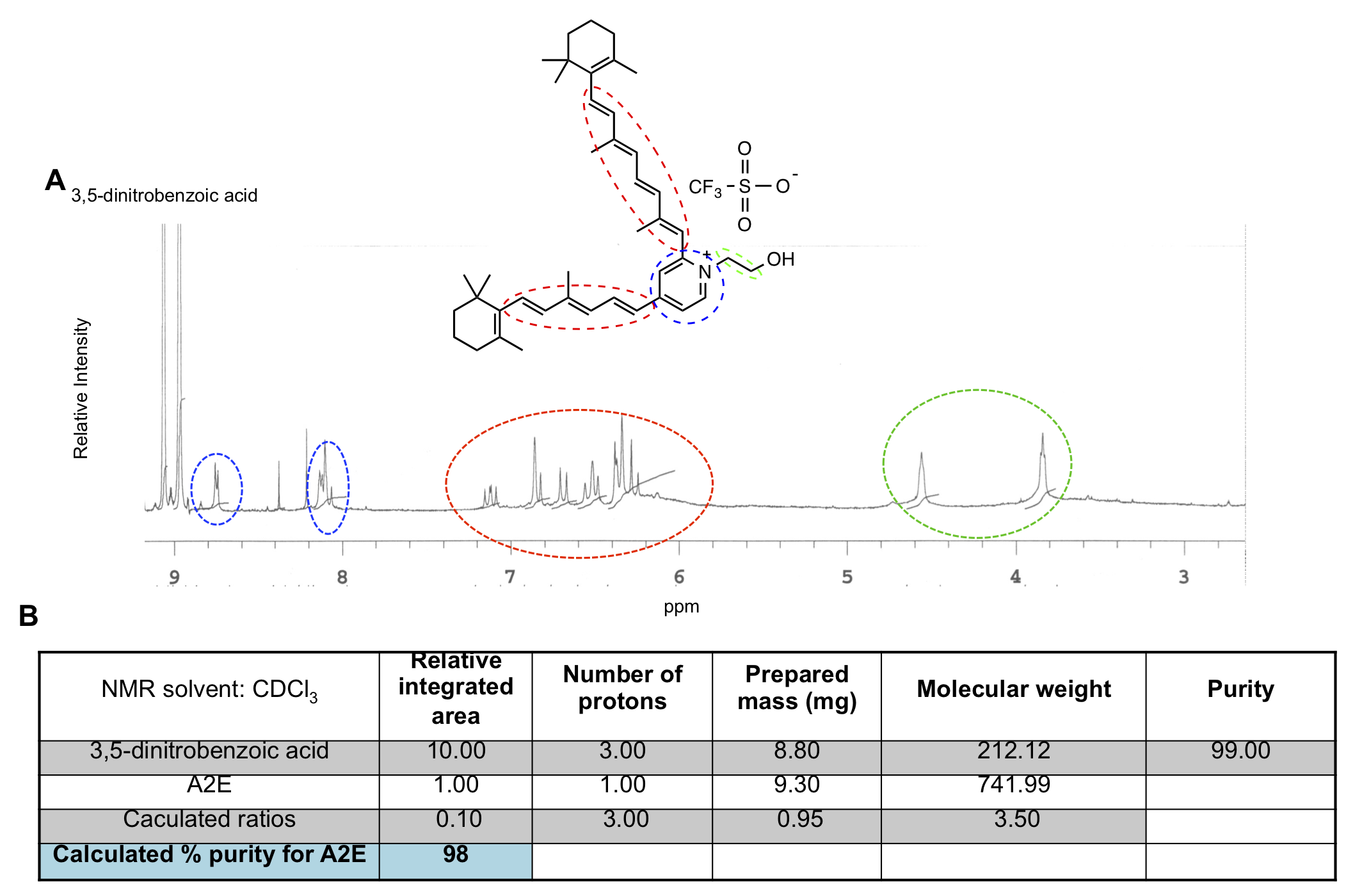
**

**Supporting Information Figure 6. A2E standard**

(**A**) Representative 300 MHz ^1^H-NMR spectrum of alkenyl (red), aromatic (blue) and aliphatic (green) protons of A2E. The corresponding protons are circled on the structure of A2E and the spectrum.

(**B**) Representative quantitative ^1^H-NMR calculation, using 3,5-dinitrobenzoic acid as a standard, to estimate the percent purity of the prepared A2E, used as an analytical standard for UPLC quantification.
